# Triple Negative Breast Cancer: A Mountain Yet to Be Scaled Despite the Triumphs

**DOI:** 10.3390/cancers13153697

**Published:** 2021-07-23

**Authors:** Qitong Wu, Sumit Siddharth, Dipali Sharma

**Affiliations:** Department of Oncology, Johns Hopkins University School of Medicine and the Sidney Kimmel Comprehensive Cancer Center at Johns Hopkins, Baltimore, MD 21231, USA; qwu38@jhmi.edu

**Keywords:** triple-negative breast cancer, signaling, chemoresistance, ABC transporters, DNA damage, metabolic reprogramming, novel therapies

## Abstract

**Simple Summary:**

Triple-negative breast cancer (TNBC) is a highly aggressive subtype of breast cancer that cannot be treated with endocrine therapy and Her2-targeted therapy. Although its prevalence among newly diagnosed breast cancers is approximately 12.7%, it accounts for 40% of breast cancer-related mortality. Higher mortality rates among TNBC is partly because of the lack of targeted therapies and the development of resistance to chemotherapy. We discuss several important mechanisms that lead to chemoresistance and focus on important pathways and biological features that can be potentially exploited to develop therapies for TNBC. TNBC is currently defined by the absence of ER, PR, and Her2 and the greatest leap for TNBC would be our ability to characterize them with the presence of ‘x’ proteins. With this review, we intend to highlight the key nodes of TNBC and push the field towards connecting the dots between key features of TNBC and novel drug(s).

**Abstract:**

Metastatic progression and tumor recurrence pertaining to TNBC are certainly the leading cause of breast cancer-related mortality; however, the mechanisms underlying TNBC chemoresistance, metastasis, and tumor relapse remain somewhat ambiguous. TNBCs show 77% of the overall 4-year survival rate compared to other breast cancer subtypes (82.7 to 92.5%). TNBC is the most aggressive subtype of breast cancer, with chemotherapy being the major approved treatment strategy. Activation of ABC transporters and DNA damage response genes alongside an enrichment of cancer stem cells and metabolic reprogramming upon chemotherapy contribute to the selection of chemoresistant cells, majorly responsible for the failure of anti-chemotherapeutic regime. These selected chemoresistant cells further lead to distant metastasis and tumor relapse. The present review discusses the approved standard of care and targetable molecular mechanisms in chemoresistance and provides a comprehensive update regarding the recent advances in TNBC management.

## 1. Introduction

Breast cancer, the most common cancer in women in the United States, accounts for 30% of all female cancers but remains curable in a large population if diagnosed at an early stage. According to SEER (Surveillance, Epidemiology, and End Results) database, approximately 276,480 new cases of breast cancer are estimated to have been diagnosed in 2020, representing 15.9% of all new cancer cases. At the same time, 42,170 women are estimated to have succumbed to the disease in 2020, representing 7% of all cancer-related deaths. Breast cancer is a heterogeneous disease that can be subdivided depending on the enrichment status of hormone receptors and human epidermal growth factor receptor 2 (HER2), respectively. Consequently, breast tumors can be categorized into four different groups: (1) hormone receptors positive with HER2 receptor-negative (Luminal A); (2) hormone receptors negative with HER2 receptor-positive (HER2 enriched); (3) positive for hormone receptors and HER2 receptor (Luminal B), and (4) triple-negative tumors, which lack the expression of any of the receptors mentioned above [1]. TNBC is infamously related to an increased rate of distant metastasis, recurrence, poorer prognosis [2,3], and a decreased overall and disease-free survival [3,4]. Yet, clinical and pathological prognostic factors related to TNBC are limited and inconsistent [5]. A higher grade of TNBC is more likely to be associated with positive EGFR (epidermal growth factor receptor), P-cadherin, and p53 expression; no enhancement of androgen receptor; and negative E-cadherin expression [3]. The overall rate of possessing deleterious BRCA1 mutation is 12 times higher in TNBC compared to other subtypes of breast cancer [2]. There are a number of subtypes under the TNBC umbrella. A broad genre includes basaloid TNBC, which expresses basal keratins; BRCA1 dysfunction TNBC; androgen-receptor pathway overexpressing TNBC; and EGFR overexpressing TNBC [1]. TNBC tumors may exhibit more than one characteristic mentioned above [1]. In 2011, molecular profiling [6] classified TNBC into six subtypes: basal-like 1 (BL1), basal-like 2 (BL2), immunomodulatory (IM), mesenchymal associated (M), mesenchymal stem-like (MSL), and luminal androgen receptor (LAR). The BL1 subtype has an enrichment of cell cycle and DNA replication components alongside an upregulated DNA damage response [7,8], whereas the BL2 subtype has elevated expression of growth factors [8]. The IM subtype is associated with immune system-related alterations such as activation of the T cells and cytokine pathways [7,8]. Both M and MSL subtypes have higher expression of genes related to cellular differentiation and cell motility [8], and the LAR subtype has heavily enriched hormone-regulated pathways [7,8].

Among different breast cancer subtypes, the mean prevalence of TNBC is approximately 12.7% [9,10], but it accounts for 40% of breast cancer-related mortality [2]. Racial disparity is very evident in TNBC, with Black, Eskimo, and Asian Indian population having a higher rate of TNBC incidence compared to the White/European population [11]. Notably, TNBC is highly prevalent in Indian women (22–43%) [12] and women with African ancestry (20–79%) [13]. Further, it has been observed that African American women have a higher incidence rate of TNBC compared to White American women [4,14,15,16,17,18,19]. In contrast to other breast cancer subtypes, women who are diagnosed with TNBC are 53% more likely to be under 40 years old [4]. TNBC disproportionately occurs in younger black women [4] who are more likely to have poorer prognostic features than older patients upon diagnosis [20]. Some of the breast cancer related risk factors such as premenopausal BMI, parity, and breastfeeding have unparalleled effects on TNBC compared to other subtypes of breast cancer [21]. The association between oral contraceptives and the risk of TNBC has been largely controversial [22,23,24,25,26,27], but a meta-analysis concluded that women who use oral contraceptives are more likely to develop TNBC compared to those who do not [28]. TNBC patients are 66% more likely to have grade III tumors when compared to other subtypes [29]. In addition, a high rate of node positivity is more prevalent among TNBC patients, even for those with small tumors. Unsurprisingly, TNBC related mortality is significantly higher compared to other breast cancer subtypes, and patients are more likely to succumb to the disease within 5 years of diagnosis [29]. 

## 2. Therapeutic Approaches for Triple-Negative Breast Cancer

According to the NCCN (National Comprehensive Cancer Network) guidelines, the standard therapeutic strategy for TNBC includes a combination of chemotherapy, surgery, and radiation therapy based on the clinicopathological features of the disease. 

### 2.1. Surgery 

Lumpectomy and mastectomy are two common surgical treatments for all breast cancers, including TNBC [30]. The presentation of TNBC on magnetic resonance imaging (MRI) is usually a unifocal mass lesion without pervasive intraductal spread [31], which makes it suitable for lumpectomy with negative resection margins [32]. Retrospective analysis investigating the correlation between overall survival (OS) and disease-free survival (DFS) with lumpectomy and mastectomy concluded that lumpectomy is a safe alternative to mastectomy for early-stage TNBC patients [30]. Interestingly, the recurrence rate does not vary among patients who underwent lumpectomy or total mastectomy [33,34]. Further, a comparative study indicated that TNBC patients with higher stage/grade tumors who undergo lumpectomy and radiotherapy have better survival than mastectomy alone [35]. Indeed, a multidisciplinary strategy involving surgery, chemotherapy, and radiation therapy yields better survival [36]. 

### 2.2. Radiation Treatment

Radiation therapy is prevalently used to reduce LRR (locoregional recurrence), which is directly associated with metastasis and TNBC-related mortality [37]. A national database study in New Zealand [37] investigated the post-surgical effect of radiation therapy in early-stage TNBC patients. They reported that TNBC patients who undergo radiation treatment after a lumpectomy have a lower 5-year recurrence rate (5.9%) compared to those who do not receive radiation treatment (32.4%). An EBCTCG (Early Breast Cancer Trialist’s Collaborative Group) study suggested that PMR (Post Mastectomy Radiotherapy) has a statistically significant positive impact for early-stage breast cancer regarding 10-year LRR, overall recurrence, and 20-year survival [38]. Efforts to enhance the impact of radiotherapy have been underway. It is worth mentioning that gold nanoparticles (AuNPs) combined with pentamidine have radio-sensitizing effects on TNBC cells. TNBC cells treated with AuNPs and pentamidine have a dose enhancement factor of 1.55 [39], implying their potential to boost up the radiotherapeutic effect.

### 2.3. Chemotherapy

The lack of hormone receptors and HER2 overexpression renders TNBC unsuitable for endocrine and HER2-targeted therapy. Therefore, cytotoxic chemotherapy is the major strategy to prevent and reduce TNBC progression and metastasis [40,41,42,43,44]. An optimal treatment plan for early-stage TNBC patients includes an assessment of treatment toxicity and benefits, as over-treatment can lead to chemo-toxicity, while under-treatment may result in recurrence and meager outcomes [45]. Chemotherapy can be administered in an adjuvant or neoadjuvant setting based on tumor characteristics [41,46,47]. Standard chemotherapy agents for TNBC include a combination of anthracyclines, alkylators, and taxanes [48]. An overview of active non-recruiting trials investigating the efficacy of combination regimens involving chemotherapy in TNBC is provided in Table 1. 

#### 2.3.1. Taxanes

Taxol, also known as paclitaxel, is an antitumor agent derived from the yew tree [49]. Docetaxel is a semi-synthetic analog of paclitaxel [49]. Both paclitaxel and docetaxel are taxanes; their mechanism of action is inhibition of microtubule depolarization, leading to hindered spindle formation. Therefore, cells are arrested in prometaphase [50,51], resulting in the inhibition of cell division. Taxanes are one of the most common chemotherapy agents for metastatic breast cancer. Weekly addition of paclitaxel to the combination therapy regimen including fluorouracil (an anti-metabolite), epirubicin (an alkylating agent), and cyclophosphamide (an immunosuppressant) show a 47% reduced recurrence rate and an 18% enhancement of 7-year disease-free survival compared to TNBC patients who do not receive paclitaxel [52]. Notably, genetic profiling analysis of TNBC subtypes revealed that the basal-like TNBC has an enriched expression of proliferation-related genes, implying its susceptibility to antimitotic agents such as taxanes [53,54]. This was confirmed by a retrospective analysis of five clinical trials [54], which indicated that the taxane-based treatment on basal-like TNBC patients leads to a clinical remission rate that is four times higher than the LAR and MSL subtypes. 

#### 2.3.2. Anthracyclines

Anthracyclines are a class of antibiotics with antineoplastic activity produced by *Streptomyces* bacterium [55]. Notable anthracyclines for breast cancer treatment are doxorubicin and epirubicin, which can interfere with DNA replication and transcription, hindering tumor cell proliferation [56]. Anthracycline-based adjuvants may decrease the risk of recurrence and breast cancer mortality by 25–30% in patients with early-stage breast cancer [57,58]. The optimal doses of doxorubicin and epirubicin as adjuvants are 60 mg/m^2^ and 100 mg/m^2^, respectively [59]. Neoadjuvant chemotherapy regimen involving doxorubicin and docetaxel shows that the pathologic complete response rate among TNBC patients is higher, compared to the non-TNBC group (17% and 3%, respectively) [60], although the overall survival and relapse-free survival is shorter. The combination therapy involving anthracyclines and taxanes has varying efficacy among patients with different TNBC subtypes. This combination treatment shows higher pathological complete response (pCR) in BL1 and MSL TNBC subtypes, while it is not optimal for patients with the LAR or BL2 subtype [51]. 

#### 2.3.3. Platinum Agents

The history of utilizing platinum agents in breast cancer therapy can be traced back to the early 1970s [61]. However, they were not utilized widely, probably due to the higher therapeutic index of other available drugs at that time [62]. Platinum agents such as cisplatin and carboplatin exert their antitumor effects by preventing the DNA strand separation, causing cell death by hindering DNA replication and transcription [63,64,65]. Of interest, it was found that cisplatin treatment inhibits growth of TNBC cells harboring BRCA1 deficiency in a dose-dependent manner [66]. Notably, cisplatin treatment is 2–3 times more potent in BRCA-deficient cells compared to BRCA1 competent cells [66]. This evidence implied that cisplatin could be advantageous to TNBC patients with aberrant BRCA1 modulation. Interestingly, cisplatin sensitivity is related to the p53 family members, ΔNp63α and TAp73, which are expressed in one-third of TNBC [67]. ΔNp63α is a transcription factor that promotes tumor cell survival by interfering with the pro-apoptotic activity of TAp73 [67,68,69]. By induction of DNA damage, cisplatin inadvertently activates the c-ABL tyrosine kinase, which phosphorylates TAp73. The phosphorylated TAp73 then binds to target genes (PUMA and NOXA), leading to apoptosis [67]. Breast cancer cells expressing ΔNp63α and TAp73 show at least 10-fold enhanced sensitivity towards platinum-based chemotherapy [67]. A phase II clinical study [70] observed that the combination therapy using cisplatin and gemcitabine (an anti-metabolite) has significant activity in patients with metastatic TNBC. Platinum-based agents, in a neoadjuvant setting, lead to a notable increase in pCR in TNBC patients compared to the placebo group whereas no improvement is noted in HER2-positive tumor [71] On the contrary, combination therapy with cisplatin, paclitaxel, and everolimus do not correlate with a significant improvement in pCR. However, the BL1 TNBC subtype demonstrates a higher sensitivity to cisplatin chemotherapy compared to other TNBC subtypes [72]. 

#### 2.3.4. Cyclophosphamide

The anti-carcinogenic effect of cyclophosphamide is less direct when compared to other therapeutics. In the liver, microsomal mixed-function oxidases convert cyclophosphamide into aldophosphamide, which is then processed by cytochrome P450 inside the tumor, yielding phosphoramide mustard and acrolein [73]. Acrolein and phosphoramide mustard are both cytotoxic, but phosphoramide is an alkylating agent that crosslinks DNA to prevent replication [74], implying that cyclophosphamide treatment is especially harmful to actively dividing cells, such as cancer cells [75]. Women with TNBC exhibit a higher rate of pCR compared to other breast cancer subtypes [76], indicating that the combined treatment of docetaxel and cyclophosphamide regimen is more suitable to treat TNBC. Neoadjuvant chemotherapy containing doxorubicin, paclitaxel, and cyclophosphamide in TNBC patients [77] show an overall pCR rate of 28%, however, the pCR rate differ significantly among different subtypes of TNBC. The pCR rate for the MSL, LAR, BL1, and BL2 subtypes are 23%, 10%, 52%, and 0%, respectively, which reiterates the importance of designing better-tailored combination regimens for different subtypes of TNBC.

## 3. Key ‘Targetable’ Mechanisms Underlying the Development of Chemoresistance in Triple-Negative Breast Cancer

At the present time, 90% of the drug failures in metastatic cancer are ascribed to chemoresistance [78]. Only 30–40% of TNBC patients show a pCR with chemotherapy alone, and they too remain susceptible to recurrence [40,79,80,81]. Often times, TNBC tumors are not explicitly differentiated [82], which is responsible for higher relapse and metastasis compared to other breast cancer subtypes. Notably, the recurrence rate of TNBC in the first 3–5 years is significantly higher in comparison to other breast cancer subtypes [83,84]. When compared to other breast cancer subtypes (82.7 to 92.5% survival), TNBC has a 77% overall 4-year survival rate [85]. Early-stage TNBC tends to show distant recurrence and poor survival within five years of diagnosis [29]. In addition, TNBCs present higher brain and visceral metastasis than hormone receptor-positive breast cancers [86]. Multiple studies show that specific hormonal therapies reduce the locoregional recurrences of luminal A/B or HER2 enriched breast cancers [87,88,89], but TNBC locoregional recurrences remain unchanged due to the lack of hormone receptors. The development of therapeutic resistance in TNBC contributes to their aggressiveness, higher recurrence, and mortality [90] and is a major obstacle in successfully treating TNBC [82]. Several mechanisms have been identified that support the development of chemoresistance in TNBC, and several nodes are being developed as potential therapeutic targets. Below we discuss few important ‘targetable’ mechanistic intersections. 

### 3.1. ABC Transporters and Drug Efflux

There are several mechanisms underlining chemoresistance in TNBC. A very classic one is related to the infamous transporter-mediated drug efflux. With the investment of ATP, the ATP-binding cassette (ABC) transporters can pump out different compounds, including different kinds of therapeutics [91]. While the family of ABC transporters is associated with the development of chemoresistance in multiple solid tumors, expression of multi-drug-resistant protein-1 (ABCC1/MRP1), multi-drug-resistant protein–8 (ABCC11/MRP8), and the breast cancer resistance protein (ABCG2/BCRP) are more likely to be enriched in TNBC compared to other breast cancer subtypes [92,93]. Induction of the hedgehog pathway in TNBC leads to drug resistance due to an overexpression of the ABC transporter [94]. Interestingly, another study showed that ABCG2 is correlated with chemoresistance in TNBC stem cells [95]. In accordance with this, Arumugam and colleagues found that inhibition of ABCG2 sensitizes TNBC cells to docetaxel [96]. Moreover, it has been noted that neoadjuvant chemotherapy leads to an up-regulation of ABCC1 in TNBC [97]. In recent years, researchers have investigated the feasibility of using ABC transporter inhibitors for chemoresistant cancer in vitro [98] and in vivo [99]. However, strategies to reverse drug resistance in breast cancer yielded inadequate efficacy in clinical trials [100]. Influences such as interstitial pressure, hypoxia, pH, and therapeutic index can contribute to decreased potency of these inhibitors in clinical trials compared with pre-clinical studies [82].

### 3.2. DNA Damage and Repair

The backbone of TNBC treatment includes DNA damaging therapeutics such as epirubicin and cyclophosphamide, which can lead to an activation of DNA damage response (DDR). Therefore, when investigating how tumor cells develop resistance to some chemotherapy regimens, their DNA repair mechanisms can be good targets. In mammalian cells, the chief upstream kinases of DDR are ATM (ataxia-telangiectasia mutated), ATR (ATM- and Rad3-related), and DNA-PKCs (DNA-dependent protein kinase) kinases [101]. When DNA double-stranded breaks (DSBs) take place, the resected DSBs stimulate ATR to activate an effector kinase, the checkpoint kinase 1 (ATR-Chk1). This step not only leads to checkpoint arrest but also induces phosphorylation of recombinase Rad51, thus triggering homologous recombination (HR) repair [102]. Subsequently, alkylating agents in TNBC patients with HR deficiency (HRD) are associated with an increased pCR rate compared to patients with other TNBC subtypes due to an exacerbated susceptibility to mutations [103]. However, Meyer and colleagues indicated that the ATR-Chk1 pathway could prevent replication stress, promoting chemoresistance of HRD TNBC to mitomycin C [104]. Therefore, DDR could be a possible underlining mechanism of chemoresistant TNBC with HRD. In addition, it has been suggested that chemoresistant TNBC could be efficiently treated with the combined therapy of ATR inhibitor and radiation [105].

MMR (Mismatch Repair) is one of the mechanisms that are responsible for correcting minor DNA aberrations such as mismatches [106]. If such a mechanism is defective, accumulation of mutations may occur, thus leading to tumorigenesis. TNBC cells with MMR-deficiency are shown to be resistant to chemotherapy that is based on antimetabolites and alkylating agents [82]. However, cells with defective MMR may have an increased tumor antigen expression, resulting in a better response to ICI (Immune checkpoint inhibitor) therapy [107]. In accordance with this, Hou and colleagues have shown that MMR deficiency in TNBC patients is strongly correlated to enrichment of PD-L1 expression [108]. These findings suggest that ICI therapy is suitable for TNBC patients with MMR deficiency [82]. O^6^-methylguanine-DNA methyltransferase (MGMT) plays a role in DNA repair by removing the mutagenic alkyl group [109]. The silencing of MGMT by epigenetic modification such as methylation of CpG islands of the promoter region is found to be prevalent in tumors [110,111]. In normal cells, such silencing may cause an increase in genome abnormality, eventually leading to tumorigenesis [112]. However, methylating MGMT in tumor cells may sensitize their response to a cytotoxic agent. Using MGMT inhibitors as a booster in chemotherapy has been investigated in patients with melanoma [113], glioblastoma [114,115], and neuroendocrine tumors [116]. Even though the research findings of MGMT methylation in breast cancer have been inconsistent [117], a study showed that methylating the MGMT promoter in chemoresistant TNBC cells results in the re-sensitization to chemotherapy, implying that MGMT could be a feasible target for chemoresistant TNBC [118]. 

### 3.3. Metabolic Reprogramming

Compared to healthy cells, cancer cells possess a more powerful metabolic capacity to meet the needs of rapid growth [82]. They generally have higher levels of oxidative phosphorylation (OXPHOS) and fatty acid β-oxidation (FAO) activities to provide energy [82]. Furthermore, MYC and MCL1 co-amplification promote chemoresistance in TNBC via the regulation of mitochondrial OXPHOS [119]. Similarly, Wang and colleagues identified a novel leptin-LEPR-JAK-STAT3-dependent FAO pathway that contributes to TNBC cell maintenance and chemoresistance [120]. Interestingly, previous studies have indicated the role of FAO in preventing anoikis (a type of programmed cell death dependent on cell attachment), which is necessary for cancer progression and metastasis [121]. Cancer cells commonly rewire metabolism to promote growth. This is called the Warburg effect [122]. The Warburg effect is critical for TNBC cells to conquer environmental changes that hinder their survival, thus, making sure that they can metastasize smoothly [82]. Since 1,6 diphosphate fructose (an intermediate of glycolysis) can promote the excretion of lactic acid, which in turn suppresses the immune system, the Warburg effect plays a role in immune escape during TNBC metastasis as well [123]. Breast cancer cells are vulnerable to oxidative stress at elevated levels [124], and it has been reported that TNBC tends to have a hypoxic phenotype [125]. Due to hypoxia, cells could be growing out of their vasculature [126]. Subsequently, the Warburg effect would be needed to compensate for this energy expense. The hypoxic tumor environment would also lead to an increase in cell senescence, possibly giving rise to a chemoresistance phenotype [127]. Indeed, an in vitro study conducted by Notte et al. demonstrated that hypoxia protects TNBC cells from paclitaxel-induced apoptosis by activation of the mTOR/JNK pathway [128]. Further, hypoxia alongside other disturbances such as altered physiological processes and DNA damage can lead to an acceleration of endoplasmic reticulum stress (ERS) [129], which has been shown to be true in TNBC [130]. Importantly, researchers also indicated that the joined forces of ERS and hypoxia signaling foster tumor progression and recurrence [130]. Martinez-Outschoorn and colleagues proposed the “*The Autophagic Tumor Stroma Model of Cancer Metabolism*,” suggesting that aggressive cancer cells put oxidative stress on stromal cells, causing them to proceed with aerobic glycolysis, thus releasing energy-rich compounds such as lactate and ketones for the survival of cancer cells [131]. This mechanism confers cancer cells the leverage to seed anywhere without a food source, thus enabling them to perpetrate metastasis [131]. Notably, an angiogenesis inhibitor could promote cancer relapse and metastasis because it causes hypoxia in the tumor-stromal micro-environment [127]. 

### 3.4. EMT and Cancer Stem Cells

Epithelial-mesenchymal transition (EMT) is a well-documented mechanism that describes the alteration of cancer cell adhesion to promote metastasis. EMT is more active in TNBC compared to other breast cancer subtypes; therefore, TNBC is more likely to metastasize [41,132,133,134]. As indicated by Neuzillet and colleagues, the TGF-β signaling pathway has been shown to promote EMT, metastatic spread, and chemoresistance [135]. Importantly, a study conducted by Asiedu and colleagues demonstrated that exposing breast cancer cells to TGF-β leads to the activation of EMT [136]. Cancer cells also acquire CSC (cancer stem cells) properties and resistance to chemotherapy. There is an undeniable connection between EMT and CSCs. The emergence of the stem-like property in tumors always came along with the activation of EMT, which enables CSCs to migrate from primary sites to distant sites [82]. The tyrosine-protein kinase MET (c-MET) pathway allows the distantly metastasized CSCs to differentiate and form metastatic colonies [82]. As reviewed by Bai and colleagues, factors that contribute to the stemness of TNBC include hypoxia [137], stromal remodeling [138], reprogramming of the *MYC/MCL1*-mediated metabolism [119], and increased activity of STAT2, Wnt/β-catenin, hedgehog, and Notch signaling pathways [139]. Tsai and colleagues investigated the molecular characteristics of recurrent TNBC and reported significant differences in the expression of some genes comparing recurrent samples from patients in early stages (IIa, IIb, and IIIa) and stage IIIc [140]. The upregulated genes in early-stage recurrence are related to cell adhesion (*KRAS*, *CSC42*, *RAC1*, and *SRGAP2*) and migration (*CDH2*), whereas WNT signaling genes are associated with late-stage recurrence [140,141]. Other notable genes include the MAPK cascade, the BDNF (brain-derived neurotropic factor) pathway, and the prostaglandin signaling pathway. Notably, an enrichment of stemness-related genes (*CD44*, *WNT4*, *WNT16*) is also observed [140]. 

Several studies [142,143,144,145,146] have indicated that the self-renewal and differentiation properties of aggressive solid tumors are related to the expansion of CSCs. Consequently, a higher ratio of CSCs to non-CSCs population is an indicator of poorer prognosis in many cancers [147,148]. When chemotherapy is administered, the majority of non-CSCs are killed, while the number of CSCs are enriched in residual tumors [149,150,151,152,153,154]. Some CSCs even enter a quiescent state, potentially contributing to future recurrence and metastasis [155,156]. Several studies reported that TNBC harbors a higher CSCs to non-CSCs ratio compared to other breast cancer subtypes [157,158]. Consistent with previous findings regarding chemoresistance and CSCs, TNBC biopsy samples show enrichment of CSCs after chemotherapy [159]. Interestingly, treatment with gemcitabine or paclitaxel activates hypoxia-inducible factors in TNBC, leading to the expression of ABC transporters, and increased signaling among the CSC population [160]. Henceforth, interrupting EMT pathway genes and/or CSC maintenance would be a promising strategy to sensitize chemoresistant TNBC cells to cytotoxic treatments [161]. 

### 3.5. Exosome and TNBC Metastasis

In recent years, there is a rising interest in exosome-related research. Exosomes are extracellular, membrane-bound vesicles that intercede intercellular communication by the transportation of regulatory molecules among cells under pathological and physiological conditions [162]. It has been indicated that exosomes secreted by tumor cells would aid in the development of drug resistance and metastasis [163]. An interesting finding reported by Ozawa and colleagues revealed that exosomes derived from chemoresistant TNBC cells can promote proliferation and bestow chemoresistance to non-malignant breast epithelial cells [164]. Another intriguing report suggested that drug efflux pumps are overexpressed in exosomes of paclitaxel resistant CAL51 cells [165]. Kavanagh and colleagues demonstrated that the therapy-induced senescent (TIS) cells release a higher amount of extracellular vesicles (EVs) in the microenvironment compared to control cells. The EVs of TIS cells contain proteins involved in ATP depletion, cell growth, apoptosis, and senescence-associated secretory phenotype factors. Other than that, those chemoresistant TIS TNBC cells also have an increased expression of ABCC1 [165]. Similarly, it has been indicated that ABCB1 confers resistance to docetaxel through EV transportation in breast [166] and prostate [167] cancer cells.

Regarding the metastatic aspect of exosomes, studies have demonstrated that exosomes secreted from tumor cells can promote the formation of pre-metastasis niche and settling a favorable microenvironment in distant metastatic sites [168] by activation of angiogenesis [169], communication with stromal cells, and extracellular matrix (ECM) remodeling [170,171]. As an example, Annexin A2 (Anxa2) is a calcium-dependent phospholipid-binding protein that participates in several cellular processes, such as adhesion, proliferation, migration, invasion, and angiogenesis [172,173]. Jeon and colleagues reported that there is an increased level of AnxA2 secretion in invasive TNBC cell lines compared to non-invasive ones [174]. Inhibiting AnxA2 in TNBC cell lines led to a reduction in their migration and invasion ability, implying that an invasive phenotype is related to the secretion of AnxA2. Importantly, a proteomic profiling database, Exocarta [175], has revealed that AnxA2 is enriched in exosomes [163]. In recent years, Maji and colleagues investigated the role of exosomal AnxA2 (exo-AnxA2) in breast cancer metastasis. They have found that exo-AnxA2 expression is significantly higher in metastatic TNBC cell lines compared to normal and pre-metastatic breast cancer cell lines [163]. Their findings demonstrated that exo-AnaxA2 encourages angiogenesis in vivo and in vitro. Additionally, Maji and colleagues observed that exosomes secreted by metastatic TNBC cells promote generation of a suitable microenvironment for metastasis. They have shown that the exo-AnxA2 is greatly influential in this process, because AnxA2-depleted exosomes is less likely to cause metastasis in vivo [163]. 

As another example regarding the effect of exosomes secreted by TNBC cells, a recent in vivo study conducted by Yuan and colleagues investigated the role of exosomes secreted by SCP28, a TNBC cell line that exhibits high bone metastatic activity [176]. They have reported that the exosome derived from SCP28 can generate a pre-metastatic niche in the bone by targeting the activity of osteoclast. By analyzing the miRNA profiles of such exosomes derived from SCP28, an enrichment of miR-21 (microRNA21) was observed [176]. Notably, it has been indicated that miR-21 expression level is enriched in TNBC cells compared to normal tissue [177], and its expression is associated with poor prognosis [178]. As reported by Fang and colleagues, inhibition of miR-21 in the TNBC cell line can lead to a pro-apoptosis effect with reduced cell proliferation, viability, and invasiveness. In accordance with this, Dong and colleagues indicated that PTEN, a pro-apoptotic factor, is down-regulated when the miR-21 expression is enhanced in TNBC cell line. Additionally, miR-21 has shown to be associated with tumorigenesis and differentiation of osteoclast [179,180]. The miR-21 exhibit its negative effects by targeting programmed cell death 4 protein (PDCD4). In the context of mediating osteoclast function and differentiation, PDCD4 suppresses c-Fos, which is involved in activation of NFATc1, a master regulator of osteoclast [179,180,181,182]. Importantly, PDCD4 has been recognized as a suppressor for tumorigenesis [183,184,185,186]. As reported by Wen and colleagues, in vitro stimulation of PDCD4 expression cannot be accomplished in TNBC when a DNA-hypermethylating agent has been introduced; yet this intervention leads to a positive result in ER+ breast cancer subtype, indicating that TNBC has a hindered expression of PDCD4 that could be pro-tumorigenesis [184]. Moreover, it has been indicated that PDCD4 can suppress metastasis in lung [187] and breast [188,189,190] cancer, suggesting that investigation of PDCD4-related pathway could have a potential in the prevention of breast cancer metastasis. Furthermore, Wu and colleagues observed that exosomal miRNA was differentially expressed between breast cancer patients with and without recurrence. Specifically, exosomal miR-150-5p, hsa-miR-576-3p, and hsa-miR-4665-5p are significantly enriched in patients with relapse, implying the impact of exosomal miRNA in recurrence, and its potential role in prognostics [191]. Taken together from evidences described above, exosomes have a significant role in drug resistance and metastasis. Since many molecules can be transported by exosomes, further exploration of this area has great potential in unveiling the mystery behind the great metastatic tendency of TNBC compared to other breast cancer subtypes. 

## 4. Therapeutic Approaches to Target Specific Pathways in Triple-Negative Breast Cancer

### 4.1. PARP Inhibitors

Approximately 40% of TNBC patients are carriers of mutated BRCA1/2 [192], implying their susceptibility to accumulate other mutations because BRCA1 and BRCA2 are crucial for repairing DNA double-strand breaks [193]. On the other hand, poly ADP ribose polymerase (PARP) has a critical role in repairing single-strand DNA damage [194]. Thus, the introduction of PARP inhibitors to cells that already have defective BRCA1/2 results in the blockade of both DNA repair mechanisms. Indeed, simultaneous interference with these two genes (BRCA1/2) and PARP results in synthetic lethality [195] as cells with BRCA2 deficiency are prone to PARP inhibitors [196]. These findings suggested that PARP inhibitors are good therapeutic candidates for TNBCs that possess defective BRCA1 or BRCA2. Olaparib is effective in breast cancer patients with BRCA mutations (BRCA1 or BRCA2) [197]. A clinical study on BRCA mutation carriers with metastatic breast cancer demonstrated that compared to standard treatment, Olaparib treatment results in longer progression-free survival (PFS) and decreased mortality [198]. Veliparib is another PARP inhibitor that has been used in conjunction with other treatments [197]. In a phase I clinical trial [199], combination therapy of veliparib, vinorelbine, and cisplatin demonstrated better progression-free survival (PFS) in patients with germline BRCA mutations (71% versus 30%), suggesting the efficiency of PARP inhibitors in alliance with other treatment [199]. Table 2 summarizes active, non-recruiting clinical trials that study different combination therapies containing PARP inhibitors in TNBC patients.

### 4.2. Angiogenesis Inhibitors

Tumor angiogenesis is predominantly mediated by proliferative cytokines such as vascular endothelial growth factors (VEGFs) and epidermal growth factors (EGFs) [200]. Compared to other subtypes of breast cancer, TNBC has a higher microvessel density [201] and intratumoral VEGF level [202]. Studies have indicated that bevacizumab, a VEGF inhibitor, leads to an improved PFS in women with TNBC [203]. In a combined therapy setting including HER2-negative metastatic breast cancer, bevacizumab + chemotherapy is found to be superior compared to chemotherapy alone [204]. A clinical study investigated the combinatorial effect of paclitaxel + bevacizumab compared to paclitaxel alone in metastatic breast cancer patients and uncovered that the combination therapy resulted in a significant extension of PFS (11.8 months v/s 5.9 months (*p* = 0.001)) compared to those who did not receive bevacizumab [205]. Further, a meta-analysis [204] found that the combination treatment of bevacizumab plus taxane-based chemotherapy is associated with an additional 2.7 month increased PFS in advanced TNBC patients. Notably, a multicenter phase II clinical trial concluded that weekly treatment of paclitaxel and carboplatin with bevacizumab as neoadjuvant in advanced TNBC patients result in clinically favorable PFS [206]. Similarly, another phase II clinical study [207] investigated the effect of bevacizumab and erlotinib (an EGF receptor inhibitor) maintenance therapy preceded by treatment of nab-paclitaxel and bevacizumab on mTNBC (metastatic TNBC) patients, which resulted in 9.1 months of increased PFS. Table 3 provides a list of active non-recruiting clinical trials that study the efficacy of combination therapy involving angiogenesis inhibitors in TNBC.

### 4.3. Inhibitors for the PI3K/AKT/mTOR Pathway

The PI3K/AKT/mTOR signaling pathway profoundly impacts tumor cell growth, proliferation, metabolism, invasion, and migration [197]. In TNBC, mutations in PIK3CA, AKT1, and inactivation of PTEN (Phosphatase and tensin homolog) are prevalent [197], which may activate the PI3K/AKT/mTOR pathway [208,209], implying the feasibility of related inhibitors as therapeutic options for TNBC patients. In a phase II clinical trial [210], the monotherapy of buparlisib, a PI3K inhibitor, demonstrated modest efficacy in patients with metastatic TNBC (mTNBC), and down regulation of the PI3K pathway was detected in a small group of patients that achieved tumor stabilization. Unfortuntely, buparlisib induced anxiety and depression in patients, partly owing to its high penetration rate to the blood-brain barrier [210,211]. The incompetence of buparlisib as monotherapy alongside its toxicity profile resulted in the discontinuation of its development for breast cancer treatment [210,212]. Yet, the role of buparlisib and other PI3K inhibitors as combinatorial treatment approaches has shown a promising outlook. Interestingly, the combination of buparlisib and olaparib demonstrated efficacy in BRCA-proficient TNBC patient-derived *xenografts* [213] as well as in tumors with defective BRCA [214]. These studies put forth that a combined therapy of PARP and PI3K inhibitors may prove beneficial for breast cancer with or without BRCA mutation. Multiple other inhibitors of PI3K/AKT/mTOR cascade have been investigated in clinical studies, predominantly in combination therapy regimens. A phase II clinical trial [215] revealed that a combined treatment of paclitaxel and ipatasertib (an AKT inhibitor) compared to paclitaxel alone resulted in a significantly prolonged PFS among TNBC patients with PIK3CA/AKT1/PTEN alteration compared to wild type TNBC patients. On the contrary, another phase II clinical trial [72] indicated that the combination therapy of cisplatin, paclitaxel, and everolimus (a mTOR inhibitor) did not show any meaningful improvement in CR or pCR (clinical response or pathologic complete response), and this combination therapy was associated with significant side effects. However, the combination therapy of doxorubicin, bevacizumab (angiogenesis inhibitor), and an mTOR inhibitor (temsirolimus or everolimus) exhibited an efficient overall response rate (ORR = 21%) among TNBC patients with PI3K/AKT/mTOR aberration [215]. Several clinical trials are currently investigating multiple combination regimens involving PI3K/AKT/mTOR inhibitors with other therapies. A list of active non-recruiting clinical trials of PI3K/AKT/mTOR inhibitors in TNBC patients is presented in Table 4. 

### 4.4. Inhibitors for Androgen Receptor (AR)

As a nuclear steroid hormone receptor, AR plays a vital role in regulating gene expression related to breast cancer in a tissue-specific manner [216,217]. Androgen receptor is activated by the binding of testosterone or dihydrotestosterone, leading to its translocation into the nucleus and activation of target genes. A multi-institutional study [218] indicated that AR differently impacts women with TNBC from different countries. An upregulation of AR is associated with a poorer prognosis for TNBC in Norway and India, but it is associated with optimal outcomes in Nigeria and United States [218]. The range of AR expression levels is 10–43% across different subtypes of TNBC [72,219,220]. In the LAR subtype of TNBC, nine times higher expression of AR is observed in comparison to other TNBC subtypes [221]. Expectedly, the AR signaling pathway has an indispensable role in the growth of LAR TNBC [6], rendering AR inhibitors potentially useful for this subtype. Indeed, treatment with bicalutamide, an AR antagonist, led to 12 weeks of PFS in AR-positive TNBC patients exhibiting the clinical efficacy of targeting AR [222]. AR-positive TNBC treated with enzalutamide (an AR inhibitor) also resulted in 3.3 months PFS and 17.6 months overall survival [223]. Another compound, abiraterone acetate, results in a reduction of serum androgen levels [224]. Bonnefoi and colleagues recorded a median PFS of 2.8 months and an ORR of 6.7 percent in LAR TNBC treated with abiraterone acetate and prednisone in a phase II clinical trial [225]. Besides binding to androgen, activation of AR can be accomplished in a ligand-independent manner, such as crosstalk with the PI3K pathway and the Ras-MEK-ERK pathway [226,227]. Interestingly, PIK3CA mutations are found to be prevalent in the LAR subtype, making them susceptible to the inhibitors for PI3K pathway [6,221], suggesting that combination therapy may have a better potential for this group. A clinical study (NCT03407529) is investigating the impact of alpelisib (PI3K inhibitor) plus enzalutamide in PTEN positive LAR TNBC patients, which may provide valuable information regarding the efficacy of the combined treatment [228]. 

### 4.5. Immunotherapy

Immunotherapy can be defined as a treatment strategy to sensitize the immune response that has been downregulated by the cancer cells. To prevent harmful autoimmunity, our immune system has checkpoints that keep T cells under control. These checkpoints have been found to be important targets for cancer cells. The most studied immune checkpoint receptor for breast cancer treatment includes the program death 1 receptor (PD-1) and its ligand (PD-L1). PD-1 is expressed on a number of immune cells; when PD-L1 binds to PD-1 on T cell, T cell response and proliferation ceases, resulting in a weakened immune response [229]. Since cancer cells proliferate rapidly, their average mutation rate is also high. In addition to the observed high tumor-infiltrating lymphocyte (TIL) level in TNBC, the immunogenicity of TNBC should be high [230]. Moreover, expression of PD-L1 was found in tumor cells and tumor-associated inflammatory cells. Notably, studies revealed that TNBC has an enrichment of PD-L1 compared to other breast cancer subtypes [231,232,233], which implied that ICIs could be introduced as immunotherapy that antagonizes the immunosuppressive aspect of TNBC, in turn reviving the antitumor effects of T cells [197,234]. In recent years, monoclonal antibodies targeting PD-1/PD-L1 have demonstrated high specificity to extracellular targets [235]. Avelumab is an antibody that inhibits PD-L1. TNBC patients expressing PD-L1 on immune cells had a better clinical response (44.4%) compared to patients without PD-L1 expression (2.6%) [236]. Similarly, Atezolizumab also targets PD-L1, and it has been approved to treat metastatic TNBC in combination with paclitaxel [107]. However, a recent study indicated that combination treatment of paclitaxel and atezolizumab did not elicit an improved outcome in TNBC patients compared to the placebo group [237]. Yet another study showed that the combination treatment with paclitaxel and atezolizumab resulted in an improved overall survival rate in TNBC patients with enriched PD-L1 compared to the placebo group [238]. In addition, pembrolizumab, a monoclonal antibody targeting PD-1 also showed anti-tumor activity in metastatic TNBC patients [239,240]. Cortes and colleagues found that combining pembrolizumab with chemotherapy increased the pCR in TNBC patients as compared to chemotherapy alone [241]. To date, Pembrolizumab is the only FDA-approved PD-1 inhibitor for TNBC patients. Some active clinical trials (NCT02734290, NCT03036488, NCT01676753) are currently evaluating the efficacy of pembrolizumab in combination therapy. Nivolumab is another inhibitor for PD-1 whose effects are being studied in several clinical trials (NCT02499367, NCT03414684) for TNBC. Furthermore, a combination treatment including cisplatin, gemcitabine, and HX008 (a PD-1 antibody) in metastatic TNBC patients is also under investigation (NCT04750382). A list of active non-recruiting trials of using an immunotherapeutic agent as a part of combination therapy is presented in Table 5.

**Figure 1:**
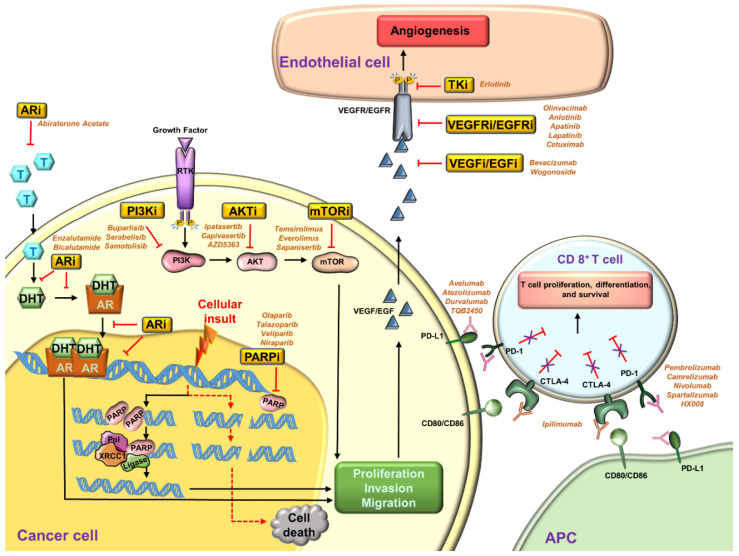
**Therapeutic approaches to target specific pathways in TNBC**. Inhibitors are mentioned in italics. (AR = androgen receptor; T = testosterone; DHT = dihydrotestosterone; PARP = poly (ADP-ribose) polymerase; XRCC1 = X-ray repair cross-complementing protein 1; Pol = polymerase); VEGF = vascular endothelial growth factor; VEGFR = VEGF receptor; EGF = epidermal growth factor; EGFR = epidermal growth factor receptor; TK = tyrosine kinase; PI3K = phosphoinositide 3-kinase; AKT = protein kinase B; mTOR = mammalian target of rapamycin; APC = antigen-presenting cell; CTLA-4 = cytotoxic T-lymphocyte-associated protein 4; CD80 = cluster of differentiation 80; CD86 = cluster of differentiation 86; PD-1 = programmed cell death protein 1; PD-L1 = programmed death-ligand 1).

On the other hand, deactivation of the T cell response can be achieved by binding cytotoxic T lymphocyte-associated protein 4 (CTLA-4) with co-stimulatory signals, such as CD80 or CD86 [242]. A recent study noted the presence of CD80 and CD86 on TNBC cells and implied their ability to down-regulate T cell activity via the CTLA-4 related pathways [243]. FDA has approved Ipilimumab (a CTLA-4 antibody) to treat advanced melanoma [51]. In treating melanoma, Ipilimumab has an ORR of 11% [244]. Bernier and colleagues demonstrated that a treatment combining CTLA-4 inhibitor and DZ-2384 (a novel microtubule-targeting compound) has significantly increased the survival in a mouse model with mTNBC [245]. Immunogenicity can be further enhanced via an epigenetic aspect because antigen processing and presentation can be affected by epigenetic silencing. Therefore, to sharpen the immunological specificity towards tumor cells, epigenetic modification factors could be a useful enhancer for immunotherapy [234]. For example, the expression of lysine-specific demethylase 1 (LSD1) was found to be inversely correlated with PD-L1 and cytotoxic T cell chemokines in clinical TNBC specimens [234]. Moreover, studies have demonstrated that a combined therapy of anti-PD-1 and LSD1 inhibitors significantly hinder TNBC tumor growth and lung metastasis compared to ICI alone [246,247]. These results imply that LSD1 inhibitors can be an effective adjuvant for TNBC immunotherapy. The target-specific pathways in TNBC are illustrated in Figure 1. 

The tumor-associated calcium signal transducer 2 (Tacstd2, also known as trophoblast cell-surface antigen 2, Trop2) is a transmembrane glycoprotein that is overexpressed as a growth-enhancing signal in many epithelial cancers [248,249]. The expression of Trop2 in TNBC patients has been found to be inconsistent. For example, Seligson and colleagues noted that up to 95% of TNBC overexpress Trop2 [250], but Khoury et al. (2019) assessed the Trop2 expression in a group of TNBC patients (n = 68), finding that only 56% of the patients were positive for Trop2 [251]. Nevertheless, an antibody-drug conjugate targeting Trop2 has shown clinical significance in TNBC patients [250,252]. This drug conjugate is also known as sacituzumab govitecan-hziy. The major composition of this is sacituzumab, the antibody of Trop2, and SN-38 (7-ethyl-10-hydroxycamptothecin), a topoisomerase inhibitor that may induce apoptosis by interfering with DNA replication [253]. A clinical study [254] reported that the sacituzumab govitecan-hziy treatment led to a prolonged PFS in mTNBC patients (5.6 months) compared to standard chemotherapy treatment (1.7 months). Sacituzumab govitecan-hziy is the first antibody conjugated drug that has been approved by the U.S. Food and Drug Administration for pretreated patients with mTNBC in advanced stage [45]. 

## 5. Conclusions and Perspectives

Despite all the progress made in segregating breast cancer into various subtypes based on their pathological and molecular characteristics, the TNBC subtype is still broadly identified on the basis of an absence of certain markers. Efforts have been underway to identify functionally significant markers for TNBC, and the greatest achievement for TNBC biology would be our ability to characterize them with the presence of ‘xy’ proteins. Identification of such functional nodes can also open up new targeted therapeutic regimens for TNBC. Another clinical challenge related to TNBC is the heterogeneity in response to standard chemotherapy regimens. It is puzzling to note that some women with TNBC respond extremely well to chemotherapy and achieve PCR while others undergo a progressive disease. Despite various advancements and accomplishments in the treatment of TNBC, chemotherapy’s paradoxical role in the generation of chemo-resistant cells leading to higher metastasis and tumor recurrence cannot be ignored. Chemoresistance can be explained as a steady decrease in the anticancer efficacy of a chemotherapeutic agent after its administration. Chemoresistance in TNBC is the product of multiple molecular alterations such as (1) activation of ABC transporters, (2) up-regulation of DNA damage response genes, (3) enrichment of cancer stem cells, and (4) activation of a proto-oncogene (which later serves as the driver gene). Chemoresistance helps cancer cells to escape therapy-induced apoptosis. Combination treatment approaches show a ray of hope to target the chemoresistant cells because the use of multiple drugs can target several signaling pathways or oncogenes responsible for driving breast tumor progression. Target-specific pathway inhibitors utilized in TNBCs, including PARP inhibitors, angiogenesis inhibitors, PI3K/AKT/mTOR inhibitors, AR inhibitors, and immunotherapy; need further exploration. TNBC’s powerful metabolic machinery combined with a hypoxic microenvironment enriches cancer stem cells, which act as lone wolves, smoothly completing an inefficient journey of metastasis to form secondary tumors, resulting in recurrence. The mechanisms underlying the development of chemoresistance in TNBC are not very well known. Therefore, a better mechanistic understanding would help in the advancement of efficient therapies to prevent metastasis and recurrence. The present review focuses on therapeutic strategies in TNBC and sheds light on the mechanisms of chemoresistance. Since most TNBC patients do not reach a PCR upon therapeutic intervention, it is imperative to decipher the molecular puzzle underlying this phenomenon. Heterogeneity in TNBC has facilitated the ongoing development of a variety of novel treatment strategies that might prove useful in the clinic and get included in the current arsenal against TNBC.

## Figures and Tables

**Table 1:** List of active non-recruiting trials of combination therapy involving chemotherapy in TNBC.

Taxanes			
Phase	Clinical Trial	Treatment	Identifier
3	A Randomized Controlled Trial of Neoadjuvant Weekly Paclitaxel Versus Weekly Paclitaxel Plus Weekly Carboplatin In Women With Large Operable or Locally Advanced, Triple-Negative Breast Cancer	Paclitaxel with or without Carboplatin as neoadjuvant following cyclophosphamide with doxorubicin or epirubicin	NCT03168880
2	A Randomized Phase II Study of Preoperative Cisplatin Versus Paclitaxel in Patients With Triple-Negative Breast Cancer: Evaluating the Homologous Recombination Deficiency (HRD) Biomarker	Either Paclitaxel or cisplatin	NCT01982448
2	Phase II Clinical Trial of Treatment With TAK-228 and TAK-117 to Inhibit Homologous Recombination (HR) Followed by Cisplatin and Nab Paclitaxel in Patients With Chemotherapy-pretreated Metastatic Triple-Negative Breast Cancer	Sapanisertib and Serabelisib with or without Cisplatin and Nab-Paclitaxel	NCT03193853
3	A Phase III, Multicenter, Randomized, Placebo-Controlled Study of Atezolizumab (Anti-PD-L1 Antibody) in Combination With Nab-Paclitaxel Compared With Placebo With Nab-Paclitaxel for Patients With Previously Untreated Metastatic Triple-Negative Breast Cancer	Nab-Paclitaxel plus placebo or Atezolizumab	NCT02425891
2	Triple-Negative First-Line Study: Neoadjuvant Trial of Nab-Paclitaxel and MPDL3280A, a PDL-1 Inhibitor in Patients With Triple-Negative Breast Cancer	Nab-paclitaxel and Atezolizumab	NCT02530489
2	A Phase II, Double-blind, Randomised, Placebo-controlled Study of the AKT Inhibitor AZD5363 in Combination With Paclitaxel in Triple-NegativeAdvanced or Metastatic Breast Cancer (PAKT).	Paclitaxel with placebo or AZD5363	NCT02423603
2	A Phase II Study of Neoadjuvant Carboplatin/Paclitaxel Followed by Dose-Dense Doxorubicin/Cyclophosphamide in Patients With Hormone Receptor Negative, HER2 Receptor Negative Breast Cancer	Carboplatin/Paclitaxel followed by Doxorubicin or Cyclophosphamide	NCT03301350
2	CADENCE: Carboplatin and Docetaxel in Neoadjuvant Treatment of ER-Negative, HER2-Negative Breast Cancer: A Co-Clinical Trial With Genoproteomic Discovery	Carboplatin plus Docetaxel	NCT02547987
1	A Phase Ib, Open-Label, Multicenter Study Evaluating the Safety and Efficacy of Ipatasertib in Combination With Atezolizumab and Paclitaxel or Nab-Paclitaxel in Patients With Locally Advanced or Metastatic Triple-Negative Breast Cancer	Ipatasertib and Atezolizumab with or without Paclitaxel or Nab-Paclitaxel; or Ipatasertib and Atezolizumab with Doxorubicin and Cyclophosphamide followed by Ipatasertib and Atezolizumab with Paclitaxe	NCT03800836
**Anthracyclines**			
2	Anti-EGFR-immunoliposomes Loaded With Doxorubicin in Patients With Advanced Triple-Negative EGFR Positive Breast Cancer—A Multicenter Single Arm Phase II Trial	Doxorubicin-loaded anti-EGFR immunoliposomes (anti-EGFR-IL-dox)	NCT02833766
2	Randomized Phase II 2 × 2 Factorial Trial of the Addition of Carboplatin +/- Bevacizumab to Neoadjuvant Weekly Paclitaxel Followed by Dose-Dense AC in Hormone Receptor-Poor/HER2-Negative Resectable Breast Cancer	Paclitaxel with or without Carboplatin and/or Bevacizumab followed by Doxorubicin and Cyclophosphamide	NCT00861705
**Cyclophosphamide**			
2	Phase II Study Of Single-dose Cyclophosphamide + Pembrolizumab In Patients With Metastatic Triple-Negative Breast Cancer	Cyclophosphamide followed by Pembrolizumab	NCT02768701
2	A Phase II Study of Neoadjuvant Carboplatin/Paclitaxel Followed by Dose-Dense Doxorubicin/Cyclophosphamide in Patients With Hormone Receptor Negative, HER2 Receptor Negative Breast Cancer	Low dose weekly Carboplatin/Paclitaxel followed by dose-dense Doxorubicin/Cyclophosphamide	NCT03301350
2/3	Randomized Phase II/III Study of Individualized Neoadjuvant Chemotherapy in ‘Triple-Negative’ Breast Tumors	Doxorubicin plus Cyclophosphamide or Carboplatin plus Thiotepa, or Doxorubicin plus Cyclophosphamide with Carboplatin and Thiotepa	NCT01057069
**Platinum agents**			
2	Phase II Trial of Neoadjuvant Chemotherapy With Carboplatin and NAB-Paclitaxel in Patients With Locally Advanced and Inflammatory Triple-Negative Breast Cancer	Carboplatin once, followed by weekly Nab-Paclitaxel	NCT01525966
2	A Randomized Phase II Trial of Carboplatin With or Without Nivolumab in First-line Metastatic Triple-negative Breast Cancer	Carboplatin with or without Nivolumab	NCT03414684
1/2	A Single-armed Multicenter Phase Ib/II Study of HX008 (a Recombinant Humanized Anti-PD-1 Monoclonal Antibody) Combined With GP Regimen as the First-line Treatment in Patients With Metastatic Triple-Negative Breast Cancer	HX008 with Cisplatin and Gemcitabine	NCT04750382
2	Pilot Study of Carboplatin, Nab-Paclitaxel and Pembrolizumab for Metastatic Triple-Negative Breast Cancer	Carboplatin, Nab-paclitaxel, and Pembrolizumab	NCT03121352

**Table 2:** List of all the active non-recruiting clinical trials of PARP inhibitors in TNBCs.

Phase	Clinical Trial	Treatment	Identifier
1	A Phase I, Open-Label Study to Assess the Safety and Tolerability of KU-0059436 in Combination With Carboplatin, KU-0059436 in Combination With a Paclitaxel/Carboplatin T/C Doublet and KU-0059436 in Combination With Paclitaxel in the Treatment of Patients With Advanced Solid Tumours	Olaparib with Carboplatin and/or Paclitaxel	NCT00516724
2	Phase 1/2 Clinical Study of Niraparib in Combination With Pembrolizumab (MK-3475) in Patients With Advanced or Metastatic Triple-Negative Breast Cancer and in Patients With Recurrent Ovarian Cancer	Niraparib plus Pembrolizumab	NCT02657889
2	Phase II Multicenter Study of Durvalumab and Olaparib in Platinum tReated Advanced Triple-Negative Breast Cancer	Olaparib with or without Durvalumab	NCT03167619
2	A Phase II, Open Label, Randomised, Multi-centre Study to Assess the Safety and Efficacy of Agents Targeting DNA Damage Repair in Combination With Olaparib Versus Olaparib Monotherapy in the Treatment of Metastatic Triple-Negative Breast Cancer Patients Stratified by Alterations in Homologous Recombinant Repair (HRR)-Related Genes (Including BRCA1/2) (VIOLETTE).	Olaparib alone or with Ceralasertib or Adavosertib	NCT03330847
1	A Phase I, Open-Label Study to Assess the Safety and Tolerability of KU-0059436 in Combination With Carboplatin, KU-0059436 in Combination With a Paclitaxel/Carboplatin T/C Doublet and KU-0059436 in Combination With Paclitaxel in the Treatment of Patients With Advanced Solid Tumours	Olaparib with Carboplatin or Paclitaxel, or Olaparib with Carboplatin and Paclitaxel	NCT00516724
N/A	An Open Label, Pilot Study of Veliparib (ABT-888) and Lapatinib (Tykerb) in Patients With Metastatic, Triple-Negative (ER, PR, and HER-2 Negative) Breast Cancer	Veliparib and Lapatinib	NCT02158507
2	Phase II Randomized Placebo-Controlled Trial of Cisplatin With or Without ABT-888 (Veliparib) in Metastatic Triple-Negative Breast Cancer and/or BRCA Mutation-Associated Breast Cancer, With or Without Brain Metastases	Cisplatin with or without Veliparib	NCT02595905
1	An Open, Non-randomised, Multi-centre Phase I Study to Assess the Safety and Efficacy of Fluzoparib Given in Combination With Apatinib in Patients With Recurrent Ovarian Cancer or Triple-Negative Breast Cancer	Fluzoparib and Apatinib	NCT03075462

**Table 3:** List of active non-recruiting trials of combination therapy involving angiogenesis inhibitors in TNBCs.

Phase	Clinical Trial	Treatment	Identifier
2	Women’s Triple-Negative First-Line Study: A Phase II Trial of Liposomal Doxorubicin, Bevacizumab and Everolimus (DAE) in Patients With Localized Triple-Negative Breast Cancer (TNBC) With Tumors Predicted Insensitive to Standard Neoadjuvant Chemotherapy	Doxorubicin, Bevacizumab, and Everolimus	NCT02456857
1	A Phase 1b, Open-Label, Safety and Tolerability Study of TTAC-0001 in Combination With Pembrolizumab in Patients With Metastatic Triple-Negative Breast Cancer	Olinvacimab and Pembrolizumab	NCT03720431

**Table 4:** List of active non-recruiting trials of PI3K/AKT/mTOR in TNBC.

Phase	Clinical Trial	Treatment	Identifier
3	A Phase III, Double-blind, Placebo-controlled, Randomized Study of Ipatasertib in Combination With Atezolizumab and Paclitaxel as a Treatment for Participants With Locally Advanced Unresectable or Metastatic Triple-Negative Breast Cancer.	Paclitaxel with Atezolizumab and placebo, or Paclitaxel with Ipatasertib and placebo, or combination of Paclitaxel, Atezolizumab, and Ipatasertib, or Paclitaxel with two placebos.	NCT04177108
3	A Double-Blind, Placebo-Controlled, Randomized Phase III Study of Ipatasertib in Combination with Paclitaxel as a Treatment for Patients with PIK3CA/AKT1/PTEN-Altered, Locally Advanced or Metastatic, Triple-Negative Breast Cancer or Hormone Receptor-Positive, HER2-Negative Breast Cancer	Paclitaxel with Ipatasertib or placebo	NCT03337724

**Table 5:** List of active non-recruiting trials involving immunotherapeutic agents in TNBC.

Phase	Clinical Trial	Treatment	Identifier
3	Adjuvant Treatment for High-risk Triple-Negative Breast Cancer Patients with the Anti-PD-l1 Antibody Avelumab: A Phase III Randomized Trial. Sponsor: Dipartimento di Scienze Chirurgiche, Oncologiche e Gastroenterologiche, Università di Padova	Surgery with or without Avelumab as adjuvant	NCT02926196
1/2	A Pilot and Phase II Study to Assess the Safety, Tolerability and Efficacy of Pembrolizumab Plus Chemotherapy in Metastatic Triple-Negative Breast Cancer Patients	Pembrolizumab with either Paclitaxel or Capecitabine	NCT02734290
3	A Phase III, Randomized, Double-blind Study to Evaluate Pembrolizumab Plus Chemotherapy vs. Placebo Plus Chemotherapy as Neoadjuvant Therapy and Pembrolizumab vs. Placebo as Adjuvant Therapy for Triple-Negative Breast Cancer (TNBC)	Pembrolizumab or placebo before and after Carboplatin and Paclitaxel	NCT03036488
1	A Phase 1b Trial of the Cyclin-dependent Kinase Inhibitor Dinaciclib in Combination With Pembrolizumab in Patients With Advanced Breast Cancer and Assessment of MYC Oncogene Overexpression	Dinacicilib and Pembrolizumab	NCT01676753
3	A Phase III Randomized Study to Investigate the Efficacy and Safety of Atezolizumab (Anti-PD-L1 Antibody) in Combination With Neoadjuvant Anthracycline/Nab-Paclitaxel-Based Chemotherapy Compared With Placebo and Chemotherapy in Patients With Primary Invasive Triple-Negative Breast Cancer	Atezolizumab plus Nab-Paclitaxel followed by Atezolizumab plus Doxorubicin and Cyclophosphamide or placebo plus Nab-Paclitaxel followed by placebo plus Doxorubicin and Cyclophosphamide	NCT03197935
2	Randomized Phase 2 Study of Neoadjuvant Chemotherapy, Carboplatin and Paclitaxel, With or Without Atezolizumab in Triple-Negative Breast Cancer (TNBC)	Carboplatin and Paclitaxel with or without Atezolizumab before surgery	NCT02883062
2	A Phase II Trial of Atezolizumab (Anti-PD-L1) With Carboplatin in Patients With Metastatic Triple-Negative Breast Cancer	Carboplatin with or without Atezolizumab	NCT03206203
3	Neo-Adjuvant Study With the PD-L1-directed Antibody in Triple-Negative Locally Advanced Breast Cancer Undergoing Treatment With Nab-paclitaxel and Carboplatin	Carboplatin and Nab-Paclitaxel with or without Atezolizumab as neoadjuvant	NCT02620280
1/2	Single Arm Neoadjuvant Phase I/II Study of MEDI4736 (Anti-PD-L1 Antibody) Concomitant With Weekly Nab-paclitaxel and Dose-dense Doxorubicin/Cyclophosphamide (ddAC) Chemotherapy for Clinical Stage I-III Triple-Negative Breast Cancer	Durvalumab followed by Nab-Paclitaxel, Docetaxel, and Cyclophosphamide	NCT02489448
2	Adaptive Phase II Randomized Non-comparative Trial of Nivolumab After Induction Treatment in Triple-negative Breast Cancer (TNBC) Patients: TONIC-trial	Nivolumab alone, or Nivolumab with Doxorubincin or Cyclophosphamide, or Cisplatin, or Radiation therapy	NCT02499367
2	A Randomized Phase II Trial of Carboplatin With or Without Nivolumab in First-line Metastatic Triple-negative Breast Cancer	Carboplatin with or without Nivolumab	NCT03414684
